# Assessment of Evidential Value Requires More than a Single Data Point

**DOI:** 10.1007/s10508-020-01836-2

**Published:** 2020-09-21

**Authors:** Roland Imhoff

**Affiliations:** Social and Legal Psychology, Department of Psychology, Johannes Gutenberg University Mainz, Binger Str. 14-16, 55122 Mainz, Germany

In his contribution, Sakaluk (2020) provides an important lesson for sex research. Embracing and adopting practices of an open, transparent, and solid science constitutes an important developmental goal for sex research, and Sakaluk does a great job of not only correcting the scientific record (by qualifying the evidential value of a recently published paper), but by tutoring and explaining the different techniques to assess evidential value. Many of the described techniques are extremely helpful tools to not just evaluate empirical contributions in the peer-review process, but also after the fact: Whole research programs and effects can be scrutinized post-peer review for their robustness, for the available evidence of evidence. Importantly, these have to be research programs, not single studies.

One critical aspect of the current state of sex research, I will thus argue, is glaringly missing from Sakaluk’s analysis, one that increasingly seems like the elephant in the room: the overreliance on single-study publications in sex research. Many fields of basic research (cognitive psychology, social psychology, personality psychology) reserve single-study papers for contributions whose empirical parts required such effort that it seems unreasonable to expect a second study (large-scale data, sample from hard-to-reach and underrepresented populations, extensive and expensive data collection as in biological studies). In contrast, single studies continue to be the norm rather than the exception in the flagship journal of sex research, the *Archives of Sexual Behavior*.

Single-study empirical papers with quantitative analyses have remained the prototypical type of article over the past five years, whereas multi-study papers are a rare species and make up barely 12% of all quantitative papers (Table 1 for frequencies of different article types; Fig. 1 for a more visual impression; raw data with coding for each article available at https://osf.io/jqm3k/). This overreliance on single studies is in my perspective one of the larger roadblocks on our way toward a more solid sexual science. I will briefly explain why I believe this to be the case. While it certainly is always nice to have more rather than less data, there are several more pressing reasons for adopting a policy that makes single-study papers the exception rather than the rule. I will briefly mention three downsides of single study papers that I see as most relevant. First, they maximize researchers’ degrees of freedom and the danger of false positives. Second, directly following up on Sakaluk (2020), single-study papers make many of the more formalized statistical tests for evidential value impossible as these rely on the distribution of statistical anomalies across independent studies. Finally, single-study contributions undermine the critical test of conceptual and theoretical patterns across instantiations of research designs. They confound theory with auxiliaries.

**Table 1:** Articles type count and percentage per year for *Archives of Sexual Behavior*, Volumes 45 to 49†

	2016 (Vol. 45)		2017 (Vol. 46)		2018 (Vol. 47)		2019 (Vol. 48)		2020 (Vol. 49)†	
	*n*	%	*n*	%	*n*	%	*n*	%	*n*	%
Non-empirical	28	13.6	68	27.1	36	15.9	83	34.7	39	20.1
Qualitative single study	10	4.9	11	4.4	29	12.8	29	12.1	16	8.2
Quantitative single study	144	69.9	132	52.6	137	60.4	97	40.6	115	59.3
Multiple studies	18	8.7	24	9.6	16	7	17	7.1	12	6.2
Review	4	1.9	14	5.6	5	2.2	10	4.2	5	2.6
Meta-analysis	2	1	2	0.8	4	1.8	3	1.3	7	3.6

^†^ Only Issues 1 through 6 included for 2020. Non-empirical papers include (predominantly) Commentaries and Letters to the Editor. Mixed-method papers (qualitative and quantitative analyses) coded as multiple studies. Table with full coding available at https://osf.io/jqm3k/

**Fig. 1:**
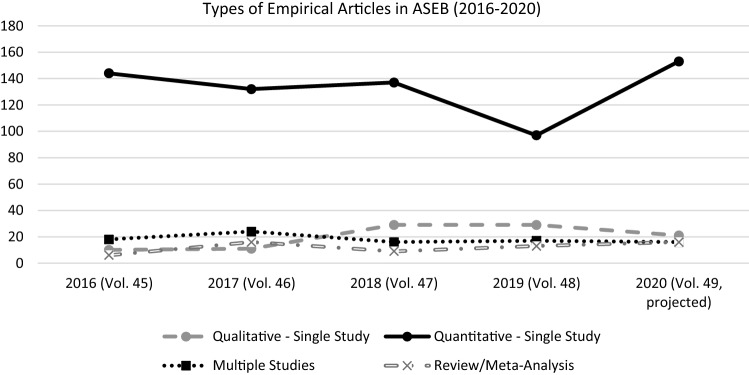
Types of empirical articles published between 2016 (Vol. 45, Issue 1) and 2020 (Vol. 49, Issue 6) in the *Archives of Sexual Behavior*. *Note*. Numbers for 2020 (Vol. 49) projected from first six issues by multiplying by 1.33

## Danger of False Positives

Psychological science has witnessed an ongoing debate about the problem of researcher-based degrees of freedom and the danger of a false positive psychology (Simmons, Nelson, & Simonsohn, 2011). The basic notion here is that there is no single correct way to analyze data, but many defensible approaches. Researchers have the liberty to pick Bayesian or frequentist approaches. There are good reasons to exclude some participants or some responses (and an infinite variety of which criteria to apply), but it is also justifiable to keep all data. It often makes a lot of sense to include covariates in critical analyses to control for theoretically uninteresting variance, but there is no clear rule when to do so and which variables to include. Likewise, there is no clear golden rule how many participants or observations to sample, but many justifiable solutions, depending on availability of resources, response rates, experience and expected effect size. For all of these decisions, one could argue, one decision is as justifiable as the other. This in and of itself is not a problem, but the state of the world: There is flexibility in deciding on data analysis. The problem arises when the decisions are taken contingent on the data. It arises when researchers continue to collect data as the effect is not there yet, when covariates are included as they make the results look better, when response times are trimmed as the researcher realized the long trials destroyed the effect, when an outlier destroyed an otherwise perfectly plausible data pattern, when one analysis proved superior to the other in teasing out the effect. Such strategies constitute an extreme case of multiple testing, increasing false positive rates at a nominal alpha of 5% to well over 50% (Simmons et al., 2011).

Importantly, these decisions are typically not made with bad intent. They do not reflect on researchers’ evil plans to claim spurious effects out of nothing. Many of these decisions make perfect sense–in hindsight. As Feynman (1974) pointed out in his remarks on cargo cult science: “The first principle is that you must not fool yourself — and you are the easiest to fool” (p. 12). To discipline one’s own analytical strategy and reduce the danger of fooling oneself into believing in an effect that is likely not robust, many have pointed to the merits of preregistration. Preregistration is the clear decision for a rationale how exactly to analyze data, how many participant to sample and how to treat outliers (among other things; see https://aspredicted.org for an intuitive and low-threshold initiation into the logic and practice of preregistration). Preregistered studies are more trustworthy than non-preregistered studies not because their analytical strategies are better or smarter, but because these were decided on a priori, before collecting the data and not in light of and influenced by the data. For those skeptical about preregistration, it is always a good intellectual game to consider the following: In 2000, prospective preregistration of clinical trials became required. Success rates (beneficial outcome of the respective drug) for large-scale studies funded by the National Heart, Lung, and Blood Institute dropped from 57% before to 8% after the implementation of this policy (Kaplan & Irving, 2015). If you were to pick a medication, would you choose one tested before or after the mandatory preregistration? The answer is simple. If we do not assume that medicine magically stopped to be effective at the turn of the millennium, these data suggest that the proportion of positive results were grossly overestimated before mandatory preregistration.

Now, this problem could be solved through preregistration, but unfortunately, sex researchers have not been on the forefront of adopting such preregistration habits. In light of this, I argue, multiple study papers help at least attenuate the problem, they provide some error control. Arguably, in a research line composed of a series of procedurally similar studies, the analytical decisions made for the first study serve more or less as preregistrations for all following studies. As an editor or a reviewer, most readers would not accept a three-study paper in which the exclusion criterion changed from study to study, in which the results were significant with a covariate in Study 1, but only without it in Study 2, etc. Researchers could still optimize their analytical strategy to the cumulative results, but their degrees of freedom would be severely limited. And it would be a lot harder to fool oneself.

## Impossibility to Run (Most) Statistical Evidential Value Tests

Single-study papers have another undesirable feature, particularly in light of the enlightening piece by Sakaluk (2020): Most evidence statistics cannot be meaningfully applied to them. Let us ignore the (faulty) single-paper meta-analysis picked up on by Sakaluk and turn to the real detective tools. Most of them build on an expectation of the distribution of effects. Like winning the lottery once is unlikely, but will not make people doubt you, reporting a single study with a *p* = 0.049 might raise a few eyebrows, but it is not a smoking gun. It is not even particularly unlikely, given a large effect (*d* = 0.5) and small sample (*n* = 20 per cell). In this scenario, roughly 3.42% of *p*-values are expected to fall between 0.04 and 0.05. Although this drops to 1.16% for sufficiently powered studies (*n* = 100 per cell), it is still not particularly noteworthy (go to https://rpsychologist.com/d3/pdist/ to play around with the *p*-value density distribution for yourself). The anomaly becomes detectable in curious distributions of statistical values. Winning the lottery thrice will raise more legitimate doubts about the processes behind it. The R index, the distribution of *p*-values as in *p*-curve, testing whether effects have insufficient variance: All these require effects (in the plural!) to begin with. Techniques that directly target the plausibility of data (like GRIM and SPRITE) still work on single studies, but exploiting researcher degrees of freedom comes in many shapes and colors and most of them will not create data anomalies detectable by GRIM or SPRITE.

## Representative Design, Conceptual Replications, and Generalizations to Theory

Forget all these arguments for a second. Even if we lay aside all suspicions about a lack of reliability in effects and trust the published data fully and even if all (single) studies were preregistered and we had installed an effective disciplinary regime of eliminating researcher-based degrees of freedom, even then multi-study papers would have a major advantage over single-study papers. They could report comprehensive research programs that critically test a theory and establish some corroboration, rather than demonstrating a singular effect. Psychological theories are theories on the conceptual level (e.g., sexual arousal leads to disinhibition; Imhoff & Schmidt, 2014) that then has to be translated into an operationalized design and therefore introduces auxiliaries (that the chosen method does induce sexual arousal, that the measurement is a valid measure of disinhibition, etc.).

For studies that fail to produce a significant effect, researchers are usually quick to come up with explanations that exonerate the theory, but blame some auxiliaries (i.e., they engage in a Lakatos’ian defense of the theory). For example, the induction did not work or the measure produced a ceiling effect. What researchers are less quick to realize is that a significant result may also just be an effect of certain auxiliaries and does not generalize to other instantiations of the same conceptual variable (e.g., another method to induce sexual arousal or another measure for disinhibition). If the claim that a woman’s success in getting a ride increases with her bust size rests on a study with a single female model (Gueguen, 2007), it is safer to conclude that bust size increased only this woman’s success (if we believe the data at all; see Brown & Heathers, 2018). Testing the theory that female waiters receive more tips than male waiters by comparing tips received by Orlando Bloom to tips received by Hillary Clinton might make one heck of a study, but is not very informative regarding the underlying theory of a gender difference.

Brunswik (1955) spoke about the concept of representative design. If we want to make a statement about the influence of male vs. female targets or about the effect of positive vs. negative stimuli or sexually arousing vs. non-arousing stimuli, we need to sample from the population of stimuli in order to generalize to this population. This is a concept that most of us are very familiar with when it comes to research participants. These need to be randomly sampled from a population in order to make valid inferences about that population. The same, however, is true for experimental materials. If we want to make a statement about positive stimuli per se, the experiment needs to randomly sample stimuli from the population of all positive stimuli. Arguably, this extreme form is as unrealistic as it is to randomly sample participants from the (world) population. Nevertheless, the other extreme appears immediately problematic to our intuition when it comes to participants (handpicking participants for each condition), but much less so for study materials (handpicking one manipulation to induce sexual arousal or carefully selecting stimuli per condition).

As with the preregistration, multi-study papers provide no salvation here. Nevertheless, they entail the possibility to focus on a conceptual point shown in various ways with various materials. The failure to falsify a prediction can then no longer be attributed to the study materials (as these are different in each study) but to the theory. A theory that can only be corroborated with one specific experimental setup, but not with other equally justifiable ones is not much worth–unless these boundary conditions can be clearly specified and theoretically explained.

In summary, Sakaluk (2020) provides many important lessons on how to evaluate articles and research programs. Most of these, however, are blunt instruments as long as sex research as a science does not start to embrace multi-study investigations and reserve single-study papers for exceptional cases where it just did not seem feasible to provide more and richer data. Admittedly, there are single studies that might not only justify to make exceptions, but that effectively address some of the issues raised above. For instance, there is a trend toward pooling resources in addressing theoretically relevant issues with sufficient statistical power in a single large-scale study (e.g., Dang et al., in press; see also: Moshontz et al., 2018), particularly in areas where large samples are difficult to recruit for each individual lab (e.g., ManyBabies Consortium, 2020). Such ManyLabs projects typically follow preregistered analysis protocols and the evidence for evidence can be estimated by treating each consortium partner as a separate study. Nevertheless, these kinds of studies typically follow a predetermined study protocol, hence limiting the generalizability of the (very precise) effect size estimate across different instantiations of the same theoretical construct. Meta-analytical approaches (a special case of a single study) do not suffer from the same problem. They allow the inclusion of diverse approaches to the same theoretical issue and moderator analyses can elucidate whether all of these approaches produce similar effects. Meta-analyses also allow for uncovering an inflation of significant effects in the original studies and different mechanisms for bias control (for an overview, see Carter, Schönbrodt, Gervais, & Hilgard, 2019). At the same time, reliance on meta-analyses to sort it out after the fact is a maximally uneconomic solution to science.

Is it fair to demand more from researchers than we currently do (see Fig. 1)? Researchers are already under constant pressure to publish (in order to get grants, tenure or recognition) and it thus might seem unfair to morally condemn their effort to publish research that is preliminary. There are two responses to these concerns. First, the particularistic interest of an individual researcher does not always map perfectly on the more universalistic interest of science per se. The scientific community, reviewers, and editors as part of the community, however, should have different interests: the advancement of science through the publication of solid results. Prioritizing this universalistic goal over each scientist’s particularistic goals is exactly what peer review is meant to achieve. As a field, it is much more economic to separate the wheat from the chaff at the entrance level, before publication. Although we have statistical tools to detecting publication bias and notoriously unreliable effects in meta-analyses, conducting and publishing dozens of studies before correcting the record do not seem like the best way to advance science. Second, more solid (and laborious) scientific papers should not just be requested, but also rewarded. That means that ultimately the incentive structure needs to change as well. Hiring and tenure committees should prioritize quality over quantity. Most these bodies are made up of researchers, not administrative bureaucrats. So, there is really no excuse not to weigh the solidness of a research program against the number of publications. Sometimes, a single multi-study paper (or the involvement in a large collaborative project) may advance science more than a collection of single-study papers of unknown reliability. Evaluating candidates might thus mean to care for the content rather than the number of their publications. Without explicitly encouraging anyone, it might also be noted that many of the tests for evidential value are not restrained to single papers, but can be used to evaluate anomalies in an individuals’ research program. It may thus be in every researcher’s own best interest to produce maximally solid results, because unreliable, lucky or spurious findings can be detected after the fact. I am convinced that it will serve our collective and self-interest as researchers, reviewers, and editors to embrace multi-study investigations as a tool toward a solid sexual science.
